# Gene Expression Profiles Identified Novel Urine Biomarkers for Diagnosis and Prognosis of High-Grade Bladder Urothelial Carcinoma

**DOI:** 10.3389/fonc.2020.00394

**Published:** 2020-03-27

**Authors:** Yuxuan Song, Donghui Jin, Ningjing Ou, Zhiwen Luo, Guangyuan Chen, Jingyi Chen, Yongjiao Yang, Xiaoqiang Liu

**Affiliations:** ^1^Department of Urology, Tianjin Medical University General Hospital, Tianjin, China; ^2^Department of Cardiothoracic Surgery, Tianjin Medical University General Hospital, Tianjin, China; ^3^Department of Hepatobiliary Surgery, National Cancer Center/National Clinical Research Center for Cancer/Cancer Hospital, Chinese Academy of Medical Sciences and Peking Union Medical College, Beijing, China; ^4^The Second Clinical Medical School, Nanchang University, Nanchang, China; ^5^Department of Gastroenterology and Institute of Clinical Molecular Biology, Peking University People's Hospital, Beijing, China; ^6^Department of Urology, The Second Hospital of Tianjin Medical University, Tianjin, China

**Keywords:** urine, biomarker, bladder urothelial cancer, diagnosis, prognosis, TCGA, GEO

## Abstract

Bladder urothelial carcinoma (BC) has been identified as one of the most common malignant neoplasm worldwide. High-grade bladder urothelial carcinoma (HGBC) is aggressive with a high risk of recurrence, progression, metastasis, and poor prognosis. Therefore, HGBC clinical management is still a challenge. We performed the present study to seek new urine biomarkers for HGBC and investigate how they promote HGBC progression and thus affect the prognosis based on large-scale sequencing data. We identified the overlapped differentially expressed genes (DEGs) by combining GSE68020 and The Cancer Genome Atlas (TCGA) datasets. Subsequent receiver operating characteristic (ROC) curves, Kaplan-Meier (KM) curves, and Cox regression were conducted to test the diagnostic and prognostic role of the hub genes. Chi-square test and logistic regression were carried out to analyze the associations between clinicopathologic characteristics and the hub genes. Ultimately, we performed gene set enrichment analysis (GSEA), protein-protein interaction (PPI) networks, and Bayesian networks (BNs) to explore the underlying mechanisms by which ECM1, CRYAB, CGNL1, and GPX3 are involved in tumor progression. Immunohistochemistry based on The Human Protein Atlas and quantitative real-time polymerase chain reaction based on urine samples confirmed the downregulation and diagnostic values of the hub genes in HGBC. In conclusion, our study indicated that CRYAB, CGNL1, ECM1, and GPX3 are potential urine biomarkers of HGBC. These four novel urine biomarkers will have attractive applications to provide new diagnostic methods, prognostic predictors and treatment targets for HGBC, which could improve the prognosis of HGBC patients, if validated by further experiments and larger prospective clinical trials.

## Introduction

Bladder urothelial carcinoma (BC) has been identified as the ninth most common malignant neoplasm all over the world (1, 2). More than 199,000 people died of it and over 549,000 cases were newly diagnosed in 2018 (1, 2). Although the age standardized incidence and number of deaths are decreasing in the past 20 years, the number of BC incident cases is growing globally and the BC burden may ascend in the future as a result of aged tendency of population and polluted environment (3, 4). BC is classified as high-grade bladder urothelial carcinoma (HGBC) and low-grade bladder urothelial carcinoma (LGBC) based on how cancer cells histologically differ from normal bladder cells (5). HGBC is aggressive and has a high risk of recurrence, progression, metastasis and poor prognosis, while LGBC is a kind of tumor with low malignancy and comparatively good prognosis (5). In addition, treatments for HGBC and LGBC are quite different. HGBC patients should receive radical cystectomy with or without postoperative chemotherapy; LGBC patients are most commonly treated with transurethral resection of bladder tumor (6, 7). Hence, an early and accurate diagnosis of BC, particularly differential diagnosis between HGBC and LGBC, is a critical factor for clinical management of BC.

At present, cystoscopy and urine cytology are commonly acknowledged as the gold standard methods for BC diagnosis (8). However, cystoscopy may sometimes miss HGBC, particularly carcinoma *in situ* (CIS). Besides, as an invasive method, cystoscopy may cause damage to surrounding organs and even lead to tumor metastasis caused by improper human operation (9, 10). Although urine cytology is a non-invasive examination, it is costly with poor sensitivity and specificity. What's more, urine cytology is subjective and varies from different pathologists' experience. The above factors contribute to the challenges and high cost associated with BC clinical management.

Recently, many urine-based tests have been carried out to explore potential biomarkers for HGBC. However, most of these urine biomarkers lack of enough sensitivity and specificity and should be used alongside cystoscopy (11). Besides, very few of these biomarkers could be utilized to predict tumor progression, metastasis and prognosis or served as potential therapeutic targets. Therefore, powerful urine biomarkers are still required to improve the diagnosis and prognosis of HGBC.

As a consequence, we conducted a series of analyses based on gene expression profile of high-throughput sequencing data obtained from Gene Expression Omnibus (GEO) and The Cancer Genome Atlas (TCGA) in order to seek potential urine biomarkers for HGBC. In the present study, we first identified the key differentially expressed genes (DEGs) by combining GEO and TCGA datasets. Then we found that ECM1 (extracellular matrix protein 1), CRYAB (alpha B-crystallin), CGNL1 (cingulin-like 1), and GPX3 (glutathione peroxidase 3) are correlated with diagnosis, progression, metastasis and prognosis of HGBC. Ultimately, we performed gene set enrichment analysis (GSEA), protein-protein interaction (PPI) networks and Bayesian networks (BNs) to explore the underlying mechanisms by which the four hub genes are involved in tumor progression. Immunohistochemistry based on The Human Protein Atlas (THPA) and quantitative real-time polymerase chain reaction (qRT-PCR) based on urine samples were utilized to validate the hub genes and their diagnostic values. In summary, this study indicated that ECM1, CRYAB, CGNL1, and GPX3 could be served as new diagnostic and prognostic urine biomarkers for HGBC.

## Materials and Methods

### GEO Data Source

The gene expression profiling dataset of GSE68020 was obtained from GEO (http://www.ncbi.nlm.nih.gov/geo/) database. Fifty urine samples including HGBC patients (*n* = 30) and non-tumor healthy controls (*n* = 20) were evaluated for BC via urine cytology. RNA was isolated and measured by microarray (Platform: GPL10558 Illumina HumanHT-12 V4.0 expression beadchip).

### TCGA Data Source

TCGA BLCA (Bladder Urothelial Carcinoma) dataset contained normal bladder samples (*n* = 19) and BC samples (*n* = 411) which included HGBC samples (*n* = 380). The RNA-sequencing data and clinical data were downloaded from TCGA (http://tcga-data.nci.nih.gov/tcga/database).

### RNA Data Processing and Identification of Differentially Expressed Genes

We mainly used R software (v.3.5.3 and v.3.4.4: http://www.r-project.org) to analyze and deal with RNA data. To identify DEGs in GSE68020 and TCGA BLCA datasets between BC patients and non-tumor healthy controls, we utilized limma R package (12). The cut-off criteria of adjusted *P*-value (*adj. P*-value) was set as 0.05 and the criterion of Fold change was set as |logFC| ≥ 1. For the identified DEGs from GSE68020 and TCGA BLCA datasets, we generated volcano plots using limma R package. For DEGs from GSE68020, we generated a heat map using pheatmap R package.

Then, an online tool, venny 2.1 (http://bioinfogp.cnb.csic.es/tools/venny/index.html) was applied to identify overlapped DEGs in the two gene expression microarrays. The upregulated and downregulated DEGs were calculated, respectively.

### Receiver Operating Characteristic Curves for Diagnostic Value

To measure the diagnostic values of the 5 hub genes for HGBC, receiver operating characteristic (ROC) curves were plotted and area under the curve (AUC) values were also calculated. Statistical analysis was performed with GraphPad Prism 7.0. *P* < 0.05 was considered as statistically significant difference.

### Survival and Statistical Analysis

Based on TCGA BLCA dataset, univariate and multivariate Cox regression, Kaplan-Meier (KM) method and log-rank test were used to compare the influence of expression levels of the 5 hub genes on overall survival (OS) along with other clinical characteristics. Clinical characteristics included Union for International Cancer Control (UICC) stage, histological grade, pathological T (pT) stage, pathological N (pN) stage, pathological M (pM) stage, age and gender. We utilized survival and survminer R packages to perform these analyses. What' more, we also used Gene Expression Profiling Interactive Analysis (GEPIA) (http://gepia.cancer-pku.cn/) for further calculating disease free survival (DFS) with the 5 hub genes on the basis of TCGA BLCA dataset (13). The correlations between clinicopathologic characteristics and expression of hub genes were analyzed with the chi-square test and logistic regression. The cut-off values of the 5 hub genes expression were determined by their median values. *P* < 0.05 was considered as statistically significant difference.

### Gene Set Enrichment Analysis

GSEA is a computational method that assesses whether a priori defined a set of genes shows statistically significant, concordant differences between two biological states (14). In the present study, GSEA firstly generated an ordered list of all genes according to their correlation with expression of hub genes, GSEA was carried out to elucidate the significant survival difference observed between high expression and low expression groups. Gene set permutations were performed 1,000 times for each analysis. The expression level of hub genes was used as a phenotype label. To illustrate the roles of ECM1, CRYAB, CGNL1, and GPX3, we carried out GSEA to analyze the enrichment of HGBC patients in TCGA BLCA dataset. False discovery rate (FDR) <25% and nominal *P* < 0.05 were regarded as the cut-off criteria of sorting Gene Ontology (GO) functional enrichment and KEGG pathway enrichment.

### Protein-Protein Interactions Network and Module Analysis

To better understand the metabolism and molecular mechanisms of carcinoma, the functional interactions between proteins become necessary. String online server (version 11.0: http://string-db.org/) was designed and adopted to collect and integrate the information by consolidating known and predicted protein-protein association data for a large number of organisms (15). ECM1, CRYAB, CGNL1 and GPX3 were, respectively, put into the tool to construct and visualize the PPI networks about each protein. Interaction score of 0.400 was set as the threshold. Besides, Cytoscape software (Cytoscape_v.3.6.1) was applied to plot the PPI networks.

### Construction of Bayesian Networks

In order to further clarify the roles of the CRYAB, ECM1, GPX3, and CGNL1 with HGBC, we constructed BNs to dissect the complex regulatory relationships among the four hub genes. BN is a graphical model that encodes probabilistic relationships among variables of interest (16). In the present study, we allowed these items as the nodes fed into the BNs: histological grade, UICC stage, pN stage and expression of the four hub genes based on TCGA BLCA dataset. Hence, we constructed three BNs and the nodes were described as follows: (1) BN1: BC histological grade+ CRYAB + ECM1 + GPX3 + CGNL1; (2) BN2: BC UICC stage + CRYAB + ECM1 + GPX3 + CGNL1; and (3) BN3: BC pN + CRYAB + ECM1 + GPX3 + CGNL1.

The conditional likelihood of the variables given their parents is represented in a BN by using Gaussian conditional densities. Under the assumption of parameter independence, an initial BN structure is learned from the training data. From this initial network, greedy search algorithm with random restarts is performed to get the highest score posterior network to avoid local maxima. Finally, an optimized BN that maximizes the Bayesian factor is obtained using heuristic search of the network space in a specified domain. The three BNs were carried out using deal R package.

### Immunohistochemistry From the Human Protein Atlas

Immunohistochemistry was obtained from The Human Protein Atlas (THPA) (http://www.proteinatlas.org/) (17). THPA is a Swedish-based program initiated in 2003 with the aim to map all the human proteins in cells, tissues and organs using integration of various omics technologies, including antibody-based imaging, mass spectrometry-based proteomics, transcriptomics and systems biology. All the data in the knowledge resource is open access to allow researchers to freely access the data for exploration of the human proteome. The Tissue Atlas and Pathology Atlas showed the distribution of the proteins across all major tissues, organs and tumors in the human body.

We evaluated expression levels of CRYAB, ECM1, GPX3, CGNL1, and CRNN (cornulin) between normal bladder tissues and HGBC tissues from THPA. Staining intensity was scored as follows: absent staining, 0; mild staining, 1; moderate staining, 2; marked staining, 3. Percentages of positive cells were categorized as follows: <5% of positive cells, 0; 5–25%, 1; 26–50%, 2; 51–75%, 3; 76–100%, 4. For each case, the two scores were multiplied to produce a total staining score. According to the total staining scores, we divided the expression into four levels: negative (-, score 0); weakly positive (+, scores 1–4); positive (++, scores 5–8); strongly positive (+++, scores 9–12).

Differences of immunohistochemistry between normal bladder tissues and HGBC tissues were compared with Mann-Whitney U test and Fisher's Exact test. *P* < 0.05 was considered as statistically significant difference. Detailed characteristics of immunohistochemistry data are in Supplementary Table 1.

### Urine Samples in Tianjin Validation Cohort, RNA Extraction and Quantitative Real-Time Polymerase Chain Reaction (qRT-PCR)

The study was approved by the Ethics Committee of Tianjin Medical University General Hospital. All recruited participants volunteered to participate and signed informed consent before being enrolled in our study.

A total of 30 patients who were pathologically and clinically diagnosed with BC were enrolled from Tianjin Medical University General Hospital. None of the BC patients had received any surgery, chemotherapy or radiotherapy before collecting urine samples. Clinical and pathological data of patients including age, gender, tumor UICC stage and histological grade were recorded. We also enrolled 30 healthy controls matched by age and sex. Urine was collected from the healthy individuals to be used as the healthy control specimens. All the 30 BC patients and 30 controls (Tianjin validation cohort) are Asians.

A single and naturally voided midstream urine sample was obtained from all recruited participants. Approximately 50 ml of urine was collected and put on ice immediately, then the samples were centrifuged as soon as possible (not later than 1 h later) at 3,000 rpm for 7 min at 4°C. Total RNA from urine samples were extracted using RNeasy kit (Qiagen, Valencia, CA). The first chain of cDNA was synthesized by reverse transcription with TaqMan^®^ Reverse Transcription Reagents (Applied Biosystems, Grand Island, NY). GAPDH was used as internal control. The sequences of the primers were displayed in Table 1. qRT-PCR was performed using the CFX96 Touch PCR system (Bio-Rad). The relative mRNA expressions of CRYAB, ECM1, GPX3, CGNL1, and CRNN were calculated by 2^−ΔΔCt^ method. In addition, ROC curves were plotted and AUC values were calculated based on the qRT-PCR results by GraphPad Prism 7.0. *P* < 0.05 was considered as statistically significant difference.

**Table 1:** Primer sequences used to amplify target genes by quantitative real-time polymerase chain reaction (qRT-PCR).

**Gene name**	**Primer sequences**	
GPX3	Forward	5′-GAAGGCTCCCCGCCAGAT-3′
	Reverse	5′-TCAATGGTGAGGGCTCCGTA-3′
ECM1	Forward	5′-AGCACCCCAATGAACAGAAGG-3′
	Reverse	5′-CTGCATTCCAGGACTCAGGTT-3′
CRYAB	Forward	5'-TGGATAGAAGGGGGACAAGGAG-3′
	Reverse	5′-CATGGAGACTTGTGATCCGGG-3′
CGNL1	Forward	5'-TACGGTGTCAGTATTCGGGTC-3′
	Reverse	5′-GCTGGGCGTATGGGTTTTC-3′
CRNN	Forward	5′-GGGATCATCGAGGCCTTCAG-3′
	Reverse	5′-CTGGATCGTGGGGTTTCACA-3′
GAPDH	Forward	5′-AGAAGGCTGGGGCTCATTTG-3′
	Reverse	5′-AGGGGCCATCCACAGTCTTC-3′

## Results

**Figure 1:**
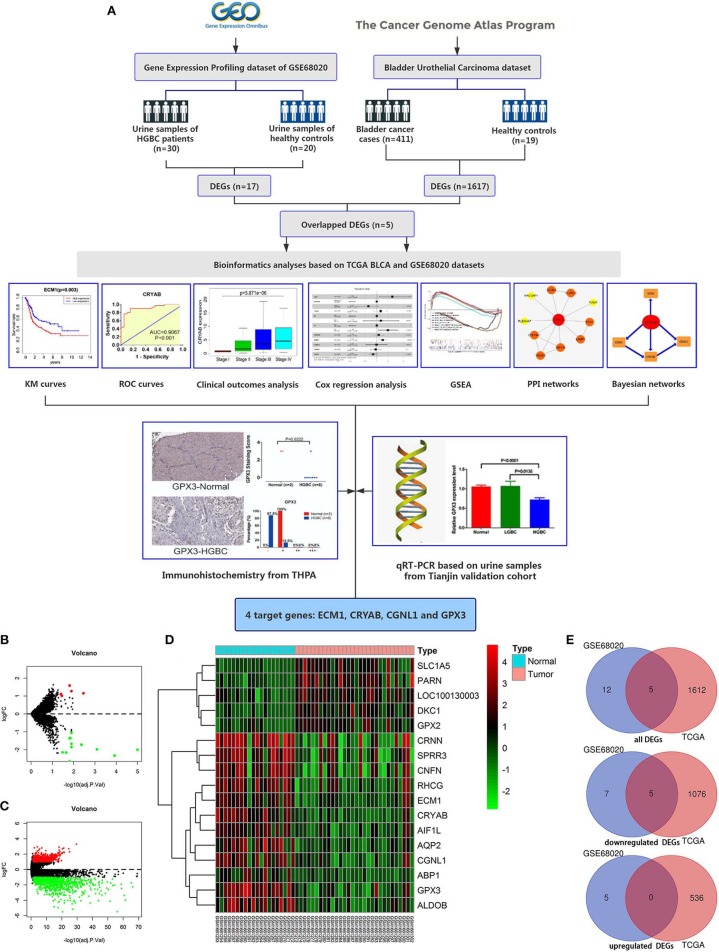
Workflow of this study and identification of differentially expressed genes (DEGs) based on GEO GSE68020 and TCGA BLCA datasets. **(A)** Workflow of this study; **(B)** Volcano plot for GSE68020 dataset; **(C)** Volcano plot for TCGA BLCA dataset; **(D)** Heat map for DEGs of TCGA BLCA dataset; and **(E)** Venn diagram for overlapped DEGs. Permissions to use the logo of GEO (Gene Expression Omnibus) have been obtained from the copyright holders of GEO. GEO, Gene Expression Omnibus; TCGA, The Cancer Genome Atlas; BLCA, Bladder Urothelial Carcinoma; KM, kaplan-Meier; ROC, receiver operating characteristic; GSEA, gene set enrichment analysis; PPI, protein-protein interaction; THPA, The Human Protein Atlas; qRT-PCR, quantitative real-time polymerase chain reaction.

Workflow for this study was displayed in Figure 1A.

### Identification of Differentially Expressed Genes

The GSE68020 dataset was processed with limma R package. According to the criteria mentioned above, a total of 17 DEGs including 5 upregulated and 12 downregulated genes were selected for further analyses as shown in the volcano plot and heat map (Figures 1B,D).

The TCGA BLCA dataset was also analyzed with limma R package. After differential expression analysis, 1617 DEGs were screened out to meet the requirements, among which 536 were upregulated and 1,081 were downregulated (Figure 1C).

To validate the reliability of DEGs, we adopted Venn diagram to obtain overlapped DEGs of the two datasets. Ultimately, 5 DEGs including CRYAB, ECM1, CGNL1, GPX3, and CRNN were confirmed to be appeared in both datasets as shown in Venn diagram (Figure 1E). All of the 5 hub genes were downregulated genes.

### Diagnostic Value of the 5 Hub Genes in GSE68020 and TCGA BLCA Datasets

**Figure 2:**
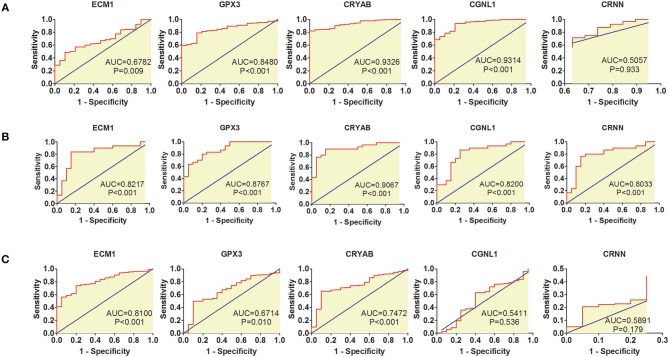
Receiver operating characteristic (ROC) curves for diagnostic values of ECM1, GPX3, CRYAB, CGNL1, and CRNN. **(A)** ROC curves of diagnostic value for high-grade bladder urothelial carcinoma (HGBC) based on TCGA BLCA dataset; **(B)** ROC curves of diagnostic value for HGBC based on GSE68020 dataset; and **(C)** ROC curves for differential diagnosis between HGBC and low-grade bladder urothelial carcinoma (LGBC) based on TCGA BLCA dataset. AUC, area under the curve.

ROC curves were applied to measure the diagnostic value of the 5 hub genes in HGBC. Based on TCGA BLCA dataset, we found that CRYAB (AUC = 0.9326, *P* < 0.001), ECM1 (AUC = 0.6782, *P* = 0.009), CGNL1 (AUC = 0.9314, *P* < 0.001), and GPX3 (AUC = 0.8480, *P* < 0.001) are effective in distinguishing HGBC tissues and normal para-carcinoma tissues (Figure 2A). However, CRNN (AUC = 0.5057, *P* = 0.933) proved to be no diagnostic capability for HGBC. Similar results were found in GEO dataset (Figure 2B).

In addition, we also evaluated whether the 5 hub genes have the potential to be used in differential diagnosis between HGBC and LGBC. We identified that CRYAB (AUC = 0.7472, *P* < 0.001), ECM1 (AUC = 0.8100, *P* < 0.001) and GPX3 (AUC = 0.6714, *P* = 0.010) can be applied in differential diagnosis between HGBC and LGBC, while CRNN (AUC = 0.5891, *P* = 0.179) and CGNL1 (AUC = 0.5411, *P* = 0.536) can't (Figure 2C).

### Survival Analysis of Hub Genes in TCGA BLCA Dataset

**Figure 3:**
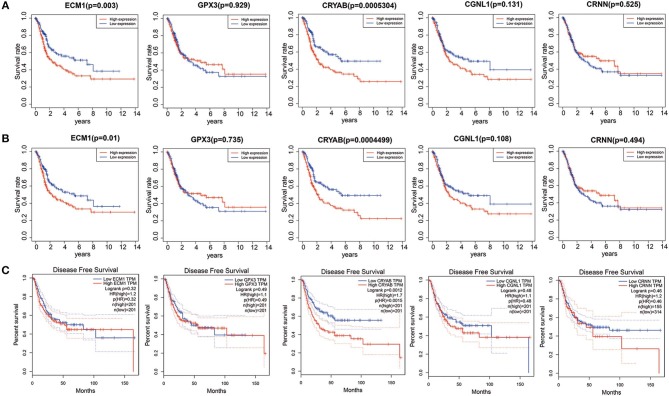
Kaplan-Meier (KM) curves for prognostic values of ECM1, GPX3, CRYAB, CGNL1, and CRNN. **(A)** Overall survival (OS) based on TCGA BLCA dataset; **(B)** OS based on high-grade bladder urothelial carcinoma in TCGA BLCA dataset; and **(C)** Disease free survival (DFS) based on Gene Expression Profiling Interactive Analysis (GEPIA).

To explore whether the 5 hub genes are associated with BC and HGBC survival time, we utilized log-rank test and drew KM curves. As shown in Figures 3A,B, we identified that higher expression levels of CRYAB and ECM1 are associated with worse OS time of BC and HGBC (*P* < 0.05), while GPX3, CGNL1 and CRNN can't influence the OS time. Furthermore, higher CRYAB expression can also lead to a poor DFS time of BC (*P* < 0.05) (Figure 3C).

### Correlations Between Expression Levels of the 5 Hub Genes and Clinical Outcomes in TCGA BLCA Dataset

**Figure 4:**
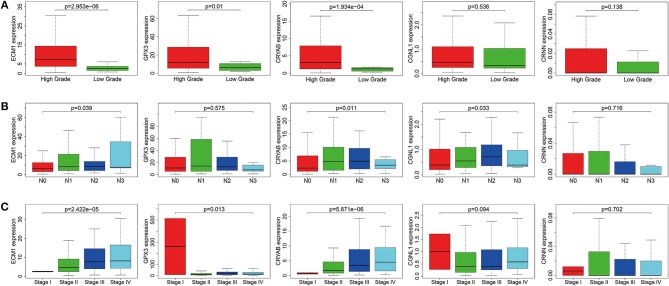
Expression levels of ECM1, GPX3, CRYAB, CGNL1, and CRNN in different clinicopathologic characteristics. **(A)** Histological grade; **(B)** pN (pathological N) stage; and **(C)** UICC (Union for International Cancer Control) stage.

To ensure whether expression levels of the 5 hub genes may influence the clinical outcomes of BC, we performed chi-square test and logistic regression. As shown in Figure 4 and Table 2, higher expression levels of CRYAB and ECM1 are observed in HGBC and advanced UICC stage (stage III and stage IV) BC (*P* < 0.05). In addition, higher expression levels of CRYAB, ECM1 and CGNL1 may predict lymph node metastasis of BC (*P* < 0.05). However, higher GPX3 expression level is an indicator for early UICC stage (stage I) (*P* < 0.05).

**Table 2:** Relationship between expression of the five DEGs and clinicopathological characteristics in BC patients from TCGA.

**Clinical characteristics**	**Total (N)**	**CRYAB expression**		***P*-value**	**ECM1 expression**		***P*-value**	**GPX3 expression**		***P*-value**	**CGNL1 expression**		***P*-value**	**CRNN expression**		***P*-value**
**(A)**		**High**	**Low**		**High**	**Low**		**High**	**Low**		**High**	**Low**		**High**	**Low**	
**Histological grade**																
High grade	380	198 (52.1%)	182 (47.9%)	**<0.001**	199 (52.4%)	181 (47.6%)	**<0.001**	196 (51.6%)	184 (48.4%)	**0.006**	190 (50.0%)	190 (50.0%)	0.383	192 (50.5%)	188 (49.5%)	0.176
Low grade	20	2 (10.0%)	18 (90.0%)		1 (00.3%)	19 (05.0%)		4 (20.0%)	16 (80.0%)		8 (40.0%)	12 (60.0%)		7 (35.0%)	13 (65.0%)	
**UICC stage**																
Stage I+II	130	43 (33.1%)	87 (66.9%)	**<0.001**	47 (12.4%)	83 (21.8%)	**<0.001**	52 (40.0%)	78 (60.0%)	**0.006**	57 (43.8%)	73 (56.2%)	0.094	71 (54.6%)	59 (45.4%)	0.189
Stage III+IV	271	158 (58.3%)	113 (41.7%)		153 (40.3%)	118 (31.1%)		148 (54.6%)	123 (45.4%)		143 (52.8%)	128 (47.2%)		129 (47.6%)	142 (52.4%)	
**pT stage**																
T1	4	0 (0.0%)	4 (100.0%)	0.052	1 (00.3%)	3 (00.8%)	0.356	2 (50.0%)	2 (50.0%)	1.000	2 (50.0%)	2 (50.0%)	1.000	3 (75.0%)	1 (25.0%)	0.623
T2+T3+T4	367	193 (52.6%)	174 (47.4%)		191 (50.3%)	176 (46.3%)		189 (51.5%)	178 (48.5%)		184 (50.1%)	183 (49.9%)		184 (50.1%)	183 (49.9%)	
**pM stage**																
No (M0)	193	78 (40.4%)	115 (59.6%)	0.988	72 (18.9%)	121 (31.8%)	0.409	77 (39.9%)	116 (60.1%)	0.215	84 (43.5%)	109 (56.5%)	0.686	91 (47.2%)	102 (52.8%)	0.485
Yes (M1)	11	5 (45.5%)	6 (54.5%)		6 (01.6%)	5 (01.3%)		7 (63.6%)	4 (36.4%)		6 (54.5%)	5 (45.5%)		4 (36.4%)	7 (63.6%)	
**pN stage**																
No (N0)	234	108 (46.2%)	126 (53.8%)	**<0.001**	109 (28.7%)	125 (32.9%)	**0.029**	116 (49.6%)	118 (50.4%)	0.430	103 (44.0%)	131 (56.0%)	**0.002**	125 (53.4%)	109 (46.6%)	0.106
Yes (N1+N2+N3)	128	84 (65.6%)	44 (34.4%)		75 (19.7%)	53 (13.9%)		69 (53.9%)	59 (46.1%)		78 (60.9%)	50 (39.1%)		57 (44.5%)	71 (55.5%)	
**Age (years)**																
≤ 60	106	39 (36.8%)	67 (63.2%)	**0.002**	48 (12.6%)	58 (15.3%)	0.271	44 (41.5%)	62 (58.5%)	**0.045**	50 (47.2%)	56 (52.8%)	0.516	58 (54.7%)	48 (45.3%)	0.246
>60	297	162 (54.5%)	135 (45.5%)		153 (40.3%)	144 (37.9%)		157 (52.9%)	140 (47.1%)		151 (50.8%)	146 (49.2%)		143 (48.1%)	154 (51.9%)	
**gender**																
Female	105	55 (52.4%)	50 (47.6%)	0.551	55 (14.5%)	50 (13.2%)	0.551	55 (52.4%)	50 (47.6%)	0.551	51 (48.6%)	54 (51.4%)	0.756	57 (54.3%)	48 (45.7%)	0.293
Male	298	146 (49.0%)	152 (51.0%)		146 (38.4%)	152 (40.0%)		146 (49.0%)	152 (51.0%)		150 (50.3%)	148 (49.7%)		144 (48.3%)	154 (51.7%)	
**(B)**		**OR (95%CI)**			**OR (95%CI)**			**OR (95%CI)**			**OR (95%CI)**			**OR (95%CI)**		
Histological grade (high vs. low)	400	9.79 (2.77, 62.14)		**0.002**	20.89 (4.27, 377.21)		**0.003**	4.26 (1.53, 15.08)		**0.011**	1.50 (0.61, 3.91)		0.386	2.38 (0.90, 7.43)		0.100
UICC stage (III+IV vs. I+II)	401	2.83 (1.83, 4.41)		**0.000**	2.30(1.49.3.54)		**0.000**	1.80 (1.18, 2.77)		**0.006**	1.43 (0.94, 2.18)		0.095	0.85 (0.56, 1.29)		0.440
pN stage (N1+N2+N3 vs. N0)	362	2.23 (1.43, 3.50)		**0.000**	1.62 (1.05, 2.52)		**0.029**	1.19 (0.77, 1.84)		0.431	1.98 (1.28, 3.09)		**0.002**	0.76 (0.49, 1.18)		0.222
pM stage (M1 vs. M0)	204	1.23 (0.34, 4.22)		0.741	2.02 (0.59, 7.22)		0.261	2.64 (0.77, 10.35)		0.132	1.56(0.45, 5.57)		0.477	0.74 (0.19, 2.54)		0.642
Age (>60 vs. ≤ 60)	403	2.06(1.31, 3.27)		**0.002**	1.28 (0.82, 2.01)		0.271	1.58 (1.01, 2.48)		**0.046**	1.16 (0.74, 1.81)		0.516	0.86 (0.55, 1.34)		0.498
Gender (male vs. female)	403	0.87 (0.56, 1.36)		0.551	0.87(0.56, 1.36)		0.551	0.87 (0.56, 1.36)		0.551	1.07 (0.69, 1.68)		0.756	0.68 (0.43, 1.06)		0.091

*A, chi-square test; B, logistic regression; DEGs, differentially expressed genes; TCGA, The Cancer Genome Atlas; UICC, Union for International Cancer Control; pT, pathological T; pN, pathological N; pM, pathological M. Bold values meant P-value < 0.05*.

In order to further confirm the prognostic value of the 5 hub genes, we performed univariate and multivariate Cox regression to calculate hazard ratios (HRs). Among 411 BC samples from TCGA BLCA dataset, 165 BC samples were enrolled in Cox regression analyses since they contained a record of complete information of UICC stage, histological grade, pT stage, pN stage, pM stage, age and gender.

Univariate Cox regression revealed that UICC stage (HR = 1.51, *P* = 0.024), pN stage (HR = 2.18, *P* = 0.003), age (HR = 2.30, *P* = 0.029) along with CRYAB (HR = 1.26, *P* = 0.026), and ECM1 (HR = 1.42, *P* < 0.001) expression status are significantly associated to OS of BC patients, while other factors including histological grade, pT stage, pM stage and gender don't have effects on OS (Table 3).

**Table 3:** Univariate and multivariate Cox analyses between expression levels of the five DEGs and patient survival based on TCGA.

**Items**	**Univariate Cox**	***P*-value**	**Multivariate Cox**	***P*-value**
Histological grade	-	0.996	-	-
UICC stage	**1.51 (1.06, 2.15)**	**0.024**	1.27 (0.85, 1.9)	0.245
pT stage	-	0.997	-	-
pM stage	2.09 (0.75, 5.80)	0.158	1.29 (0.44, 3.8)	0.641
pN stage	**2.18 (1.30, 3.66)**	**0.003**	1.97 (1.08, 3.58)	**0.026**
Age	**2.30 (1.09, 4.85)**	**0.029**	2.58 (1.18, 5.64)	**0.018**
Gender	0.59 (0.34, 1.03)	0.062	0.61 (0.34, 1.09)	0.095
CRYAB	**1.26 (1.03, 1.55)**	**0.026**	1.12 (0.83, 1.52)	0.443
ECM1	**1.42 (1.18, 1.71)**	**<0.001**	**1.44 (1.17, 1.78)**	**0.001**
CGNL1	1.19 (0.71, 1.98)	0.511	0.98 (0.52, 1.85)	0.957
CRNN	1.16 (0.86, 1.57)	0.339	0.94 (0.68, 1.3)	0.700
GPX3	0.97 (0.82, 1.15)	0.716	**0.81 (0.65, 0.99)**	**0.043**

*DEGs, differentially expressed genes; TCGA, The Cancer Genome Atlas; UICC, Union for International Cancer Control; pT, pathological T; pN, pathological N; pM, pathological M. Bold values meant P-value < 0.05*.

Multivariate Cox regression was carried out with every gene, respectively. We demonstrated that higher ECM1 expression (HR = 1.44, *P* = 0.001), lymph node metastasis (HR = 1.97, *P* = 0.026), and advanced age (HR = 2.58, *P* = 0.018) might be considered as independent poor prognostic indicators of OS. However, higher GPX3 expression might be an independent good prognostic indicator (HR = 0.81, *P* = 0.043) (Table 3).

### GSEA Revealed Biological Function of Hub Genes in BC (GO and KEGG Pathway Analysis)

To explore the underlying mechanisms by which CRYAB, ECM1, GPX3, and CGNL1 are involved in BC progression, GSEA was carried out between high expression and low expression groups on the basis of TCGA BLCA dataset. Both KEGG pathway analysis and GO functional enrichment were performed.

**Figure 5:**
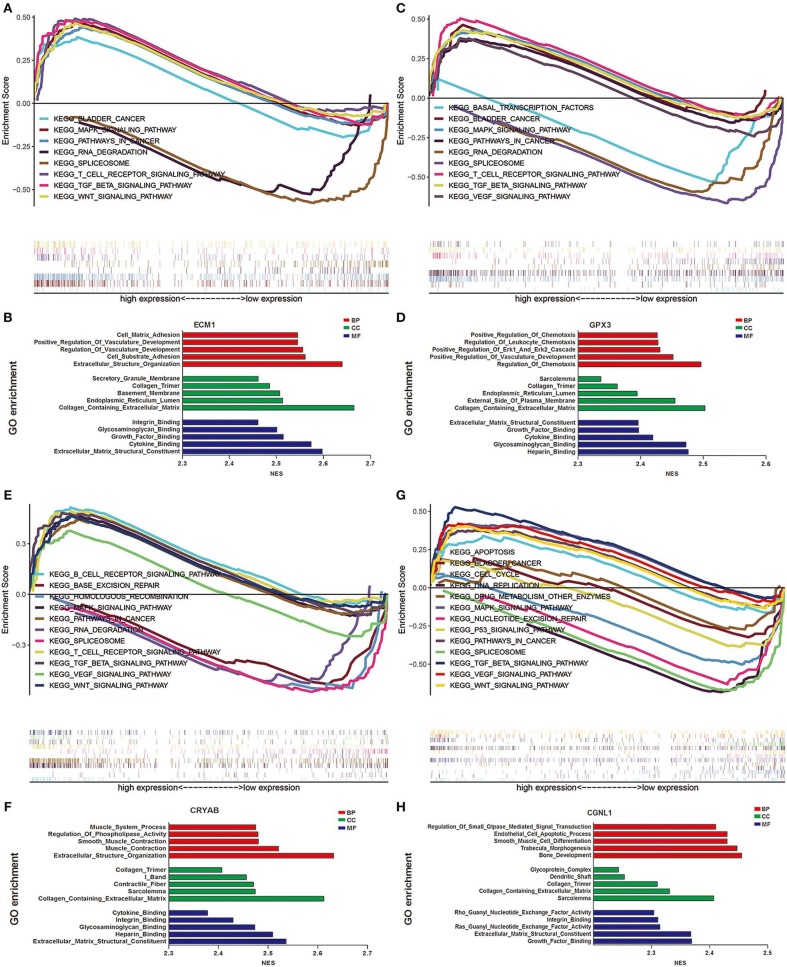
Gene set enrichment analysis (GSEA) analysis of ECM1, GPX3, CRYAB, and CGNL1. **(A)** KEGG pathway enrichment for ECM1; **(B)** Gene Ontology (GO) enrichment for ECM1; **(C)** KEGG pathway enrichment for GPX3; **(D)** GO enrichment for GPX3; **(E)** KEGG pathway enrichment for CRYAB; **(F)** GO enrichment for CRYAB; **(G)** KEGG pathway enrichment for CGNL1; and **(H)** GO enrichment for CGNL1. BP, biological process; CC, cell component; MF, molecular function; NES, Normalized Enrichment Score.

We identified pathways that are differentially activated in HGBC. Upregulation of CRYAB, ECM1, GPX3, and CGNL1 were enriched in pathways which are vital in tumorigenesis and progression, such as vascular endothelial growth factor (VEGF), TGF-β (transforming growth factor-β), Wnt and MAPK signaling pathways. Downregulation of the four genes can have effects on spliceosome (Figures 5A,C,E,G).

GO functional enrichment was also conducted and we found that the CRYAB, ECM1, GPX3, and CGNL1 are enriched in biological process (BP) including extracellular matrix structural constituent, glycosaminoglycan binding and cytokine binding. With regard to molecular function (MF), they are enriched in collagen containing extracellular matrix and collagen trimer. As for cell component (CC) analysis, they are located in extracellular structure organization and regulation of vasculature development. Based on the 3 genes, the top five significant GO terms for BP, CC, and MF are shown in Figures 5B,D,F,H.

### PPI Network Analyses and Module Functional Enrichment

**Figure 6:**
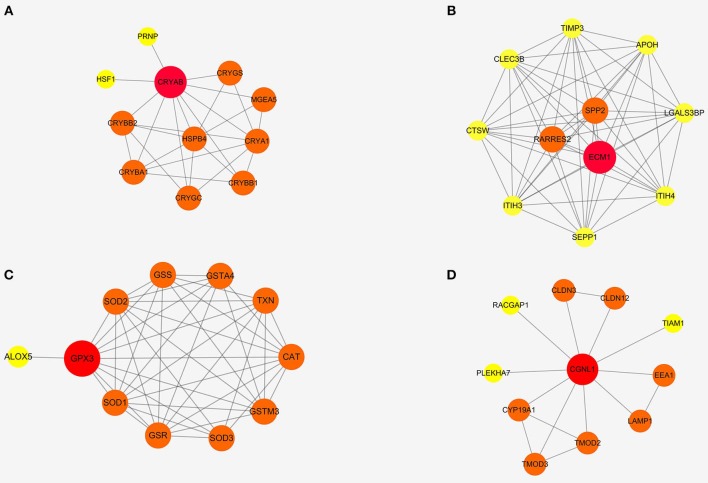
Protein-protein interaction (PPI) networks of four modules based on ECM1, GPX3, CRYAB, and CGNL1. **(A)** CRYAB module PPI network; **(B)** ECM1 module PPI network; **(C)** GPX3 module PPI network; and **(D)** CGNL1 module PPI network.

To further detect the interaction between the proteins encoded by CRYAB, ECM1, GPX3, and CGNL1, the four genes were put into String separately. Based on the String database, we constructed four PPI network modules in Figure 6. Besides, functional enrichments of the four modules were shown in Table 4.

**Table 4:** GO functional and KEGG pathway enrichments of PPI networks based on the four gene modules.

**CGNL1**			**GPX3**			**ECM1**			**CRYAB**		
**GOterm/pathway**	**Description**	**FDR**	**GOterm/pathway**	**Description**	**FDR**	**GOterm/pathway**	**Description**	**FDR**	**GOterm/pathway**	**Description**	**FDR**
**BP (GO)**											
GO:0051694	Pointed-end actin filament capping	0.0067	GO:1990748	Cellular detoxification	3.60E-14	GO:0002576	Platelet degranulation	6.94E-22	GO:0007601	Visual perception	5.15E-07
GO:0031032	Actomyosin structure organization	0.0067	GO:0098869	Cellular oxidant detoxification	3.53E-12	GO:0010466	Negative regulation of peptidase activity	7.33E-06	GO:0051260	Protein homooligomerization	0.00011
GO:0030036	Actin cytoskeleton organization	0.0135	GO:0006749	Glutathione metabolic process	9.98E-09	GO:0030162	Regulation of proteolysis	4.12E-05	GO:0003008	System process	0.00013
GO:0016338	Calcium-independent cell-cell adhesion via plasma membrane cell-adhesion molecules	0.0135	GO:0006979	Response To Oxidative Stress	3.25E-08	GO:0010951	Negative regulation of endopeptidase activity	0.00021	GO:0050877	Nervous system process	0.00018
GO:0007015	Actin filament organization	0.0172	GO:0034599	Cellular response to oxidative stress	9.65E-08	GO:0051336	Regulation of hydrolase activity	0.00059	GO:0032387	Negative regulation of intracellular transport	0.00051
**MF (GO)**											
GO:0005523	Tropomyosin binding	0.0027	GO:0016209	Antioxidant activity	7.12E-13	GO:0004866	Endopeptidase inhibitor activity	8.89E-05	GO:0005212	Structural constituent of eye lens	1.02E-19
GO:0008092	Cytoskeletal protein binding	0.0426	GO:0016491	Oxidoreductase activity	2.24E-08	GO:0030234	Enzyme regulator activity	0.0011	GO:0051082	Unfolded protein binding	0.00069
GO:0005515	Protein binding	0.0426	GO:0004784	Superoxide dismutase activity	2.19E-07	GO:0004867	Serine-type endopeptidase inhibitor activity	0.0087	GO:0042802	Identical protein binding	0.0031
—	—	—	GO:0003824	Catalytic Activity	1.87E-05	GO:0002020	Protease binding	0.0137	GO:0001540	Amyloid-beta binding	0.0074
—	—	—	GO:0043295	Glutathione binding	0.00033	GO:0008201	Heparin binding	0.0173	—	—	—
**CC (GO)**											
GO:0043296	Apical junction complex	0.00015	GO:0031970	Organelle envelope lumen	0.0016	GO:0031089	Platelet dense granule lumen	6.28E-32	—	—	—
GO:0005911	Cell-cell junction	0.00015	GO:1904813	Ficolin-1-rich granule Lumen	0.0472	GO:0005576	Extracellular region	7.36E-10	—	—	—
GO:0005923	Bicellular tight junction	0.0023	GO:0044444	Cytoplasmic part	0.0472	GO:0044421	Extracellular region part	7.40E-06	—	—	—
GO:0044430	Cytoskeletal part	0.0024	GO:0044429	Mitochondrial part	0.0472	GO:0005615	Extracellular space	4.37E-05	—	—	—
GO:0019897	Extrinsic component of plasma membrane	0.0024	GO:0044421	Extracellular region part	0.0472	GO:0031012	Extracellular matrix	0.0012	—	—	—
**KEGG**											
hsa04530	Tight junction	0.0018	hsa00480	Glutathione metabolism	1.92E-09	—	—	—	hsa04141	Protein processing in endoplasmic reticulum	0.0216
hsa05152	Tuberculosis	0.0265	hsa04213	Longevity regulating pathway-multiple species	7.82E-05	—	—	—	—	—	—
hsa04145	Phagosome	0.0265	hsa04146	Peroxisome	0.00012	—	—	—	—	—	—
—	—	—	hsa05418	Fluid shear stress and atherosclerosis	0.00038	—	—	—	—	—	—
—	—	—	hsa05014	Amyotrophic lateral sclerosis	0.0022	—	—	—	—	—	—

*Go, Gene Ontology; BP, biological process; CC, cell component; MF, molecular function; FDR, false discovery rate*.

### Construction of Bayesian Networks

**Figure 7:**
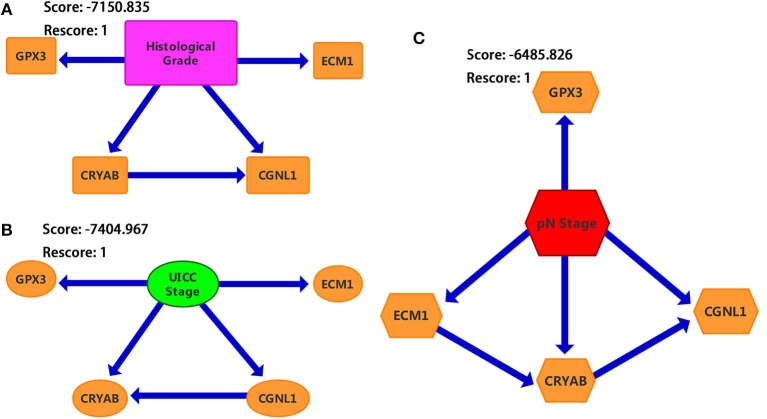
Bayesian networks (BNs) based on ECM1, GPX3, CRYAB, and CGNL1 with bladder urothelial carcinoma (BC). **(A)** Histological grade; **(B)** UICC (Union for International Cancer Control) stage; and **(C)** pN (pathological N) stage.

We constructed three BNs as described in Materials and methods section. Since chi-square test and logistic regression show that CRYAB, ECM1, CGNL1, and GPX3 are significantly associated with histological grade, UICC stage and pN stage (*P* < 0.05), we combined the four hub genes with histological grade, UICC stage and pN stage, respectively, to construct the three BNs in Figure 7. In each of BNs, an edge specified as node1→ node2 means that node2 is a direct cause of node1.

From Figure 7, we identified that CRYAB, ECM1, CGNL1, and GPX3 are all direct factors of BC histological grade, UICC stage and pN stage. This discovery is consistent with the results of chi-square test and logistic regression. It is worth noting that CGNL1 is a direct cause of CRYAB for BC histological grade as shown in BN1. In the meantime, it is interesting to note that CRYAB is a direct cause of CGNL1 for BC UICC stage as shown in BN2. Furthermore, with regard to BC pN stage, BN3 illustrated that CGNL1 is a direct cause of CRYAB and CRYAB is a direct cause of ECM1.

### Immunohistochemistry From the Human Protein Atlas

**Figure 8:**
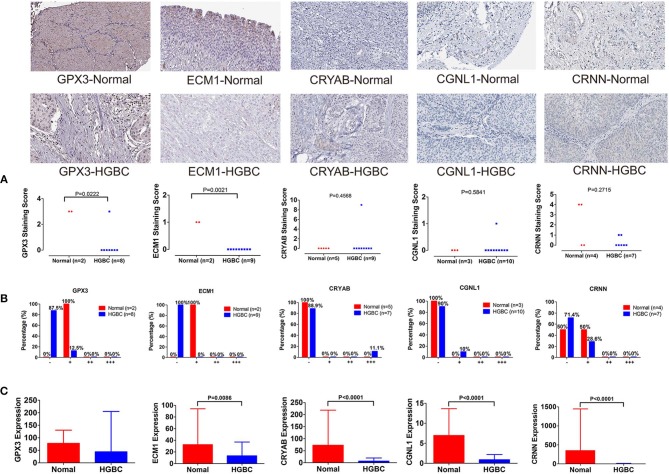
Immunohistochemistry from The Human Protein Atlas (THPA) confirmed the downregulation of GPX3 and ECM1 in high-grade bladder urothelial carcinoma (HGBC) tissues by Mann-Whitney U test (*P* < 0.05). **(A)** Mann-Whitney *U* test compared the staining scores of immunohistochemistry between normal bladder tissues and HGBC tissues; **(B)** Distributions of the four expression levels [negative (–), weakly positive (+), positive (++) and strongly positive (+ + +)] of immunohistochemistry; and **(C)** Expressions of the 5 genes in TCGA BLCA dataset.

To further validate our above findings, we evaluated expression levels of CRYAB, ECM1, GPX3, CGNL1 and CRNN between normal bladder tissues and HGBC tissues based on immunohistochemistry from THPA. As shown in Figure 8A, Mann-Whitney *U* test suggested that normal bladder tissues have higher staining scores of GPX3 (*P* = 0.0222) and ECM1 (*P* = 0.0021) than HGBC tissues. However, there is no difference for the staining scores of CRYAB, CGNL1 and CRNN between the two groups (*P* > 0.05).

In addition, we divided the expression into four levels: negative (–), weakly positive (+), positive (++) and strongly positive (+++). Distributions of the four expression levels for each gene were demonstrated in Figure 8B. Fisher's Exact test showed that normal bladder tissues have higher expression level of GPX3 (*P* = 0.016) and ECM1 (*P* = 0.001) than HGBC tissues, while no differences were found for expression levels of CRYAB, CGNL1 and CRNN (P > 0.05) (Table 5).

**Table 5:** Comparison of immunohistochemistry expression level between normal bladder and HGBC by Fisher's Exact test from The Human Protein Atlas.

**Gene name**		***N***	**Immunohistochemistry expression level**				***P*-value**
			**Negative (–)**	**Weakly positive (+)**	**Positive (++)**	**Strongly positive (+++)**	
GPX3	Normal bladder tissue	2	0	2	0	0	**0.016**
	HGBC tissue	8	7	1	0	0	
ECM1	Normal bladder tissue	2	0	2	0	0	**0.001**
	HGBC tissue	9	9	0	0	0	
CRYAB	Normal bladder tissue	5	5	0	0	0	0.439
	HGBC tissue	9	8	0	0	1	
CGNL1	Normal bladder tissue	3	3	0	0	0	0.569
	HGBC tissue	10	9	1	0	0	
CRNN	Normal bladder tissue	4	2	2	0	0	0.477
	HGBC tissue	7	5	2	0	0	

*Immunohistochemistry expression level: the expression of immunohistochemistry was divided into four levels: negative (–), weakly positive (+), positive (++) and strongly positive (+++); HGBC, high-grade bladder urothelial carcinoma. Bold values meant P-value < 0.05*.

The results of immunohistochemistry confirmed that GPX3 and ECM1 are differentially expressed between HGBC tissues and normal bladder tissues, which is consistent with the results of TCGA BLCA dataset. Figure 8C showed the expression levels of the 5 genes in TCGA BLCA dataset.

### Expression of GPX3, ECM1, CRYAB, CGNL1, and CRNN in Tianjin Validation Cohort

We recruited 30 BC patients and 30 controls from Tianjin Medical University General Hospital for further validation. Clinical characteristics of enrolled BC patients and controls in Tianjin validation cohort are displayed in Table 6. The chi-square test revealed that the patients and controls are matched for age (*P* = 0.602) and gender (*P* = 0.438). Among the 30 BC patients, 13 were LGBC patients and 17 were HGBC.

**Table 6:** Characteristics of enrolled participants from Tianjin validation cohort.

	**BC patients (*n* = 30)**	**Controls (*n* = 30)**	**chi-square**	***P-value***
**Age (years)**				
≤60	14 (46.67%)	12 (40.00%)	0.271	0.602
>60	16 (53.33%)	18 (60.00%)		
**Gender**				
Male	13 (43.33%)	16 (53.33%)	0.601	0.438
Female	17 (56.67%)	14 (46.67%)		
**UICC stage**				
Stage I	7 (23.33%)			
Stage II	8 (26.67%)			
Stage III	9 (30.00%)			
Stage IV	6 (20.00%)			
**Histological grade**				
Low grade	13 (43.33%)			
High grade	17 (56.67%)			

**Figure 9:**
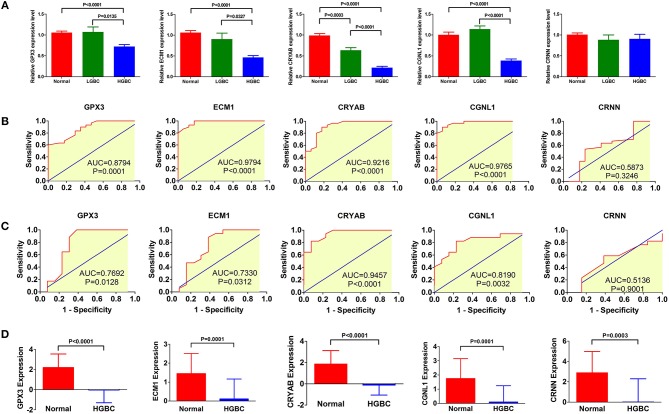
qRT-PCR of urine samples from Tianjin validation cohort. Relative expressions of GPX3, ECM1, CRYAB, and CGNL1 are significantly lower in urine of high-grade bladder urothelial carcinoma (HGBC) patients than in controls (*P* < 0.05). **(A)** qRT-PCR of urines samples from controls, low-grade bladder urothelial carcinoma (LGBC) and HGBC; **(B)** Receiver operating characteristic (ROC) curves of diagnostic value for HGBC based on qRT-PCR; **(C)** ROC curves for differential diagnosis between HGBC and LGBC based on qRT-PCR; and **(D)** Expressions of the 5 genes in GSE68020 dataset. AUC, area under the curve.

To investigate and confirm whether the five genes were detectable and altered in urine samples of BC patients compared with healthy controls, we performed qRT-PCR to detect the expression levels of GPX3, ECM1, CRYAB, CGNL1 and CRNN at mRNA level, respectively. As shown in Figure 9A, the relative expressions of GPX3, ECM1, CRYAB, and CGNL1 are significantly lower in urine of HGBC patients than in controls (*P* < 0.05), while no difference were revealed in CRNN expression (*P* > 0.05).

Figure 9B displayed the ROC curves performed to investigate the diagnostic value of the five genes for HGBC. The results suggested that expressions of GPX3 (AUC = 0.8794, *P* = 0.0001), ECM1 (AUC = 0.9794, *P* < 0.0001), CRYAB (AUC = 0.9216, *P* < 0.0001), and CGNL1 (AUC = 0.9765, *P* < 0.0001) have good predictive power for diagnosis of HGBC, indicating that they may be used as an urine biomarker for HGBC.

Figure 9C showed the ROC curves conducted to evaluate the predictive value of differential diagnosis between HGBC and LGBC. We identified that GPX3 (AUC = 0.7692, *P* = 0.0128), ECM1 (AUC = 0.7330, *P* = 0.0312), CRYAB (AUC = 0.9457, *P* < 0.0001), and CGNL1 (AUC = 0.8190, *P* = 0.0032) can be applied in differential diagnosis between HGBC and LGBC, while CRNN (AUC = 0.5136, *P* = 0.9001) can't.

The results of qRT-PCR confirmed that GPX3, ECM1, CRYAB and CGNL1 are lower in urine of HGBC patients than controls, which is consistent with the results of GSE68020 dataset. Figure 9D showed the expression levels of the 5 genes in GSE68020 dataset.

## Discussion

Accumulating evidence suggests that bioinformatics analysis would be an effective method to find novel molecular biomarkers in early diagnosis, therapeutic process monitoring and prognostic evaluation of cancer (18). Although previous investigations have identified various biomarkers for BC, most of biomarkers have not been applied in clinical practice for their inconsistent performance in terms of specificity and/or sensitivity (19). Besides, very few of the studies have focused on biomarkers for HGBC. In the present study, TCGA BLCA dataset, a large-scale prospective cohort research, and GSE68020 dataset from GEO were exploited in order to explore potential urine biomarkers for HGBC.

Our findings indicated that CRYAB, ECM1, CGNL1, and GPX3 are effective urine biomarkers for HGBC diagnosis, of which CRYAB, ECM1 and GPX3 are also urine biomarkers for differential diagnosis between HGBC and LGBC. Besides, CRYAB, ECM1, and GPX3 are potential urine prognostic factors for HGBC; ECM1 and GPX3 might be considered as independent prognostic indicators for HGBC. According to clinicopathologic characteristics, we identified that CRYAB, ECM1, GPX3, and CGNL1 may predict histological grade, UICC stage and lymph node metastasis. In order to further validate these findings, we extracted immunohistochemistry of normal bladder tissues and HGBC tissues for these hub genes from THPA. In addition, we also performed qRT-PCR of these hub genes based on the urine samples from 30 BC patients and 30 controls in Tianjin validation cohort. The results confirmed the different expression levels of CRYAB, ECM1, CGNL1, and GPX3 between HGBC patients and controls, and their diagnostic values were also proved. The above findings could provide new diagnostic methods, prognostic predictor and treatment targets for HGBC, which could improve the prognosis of HGBC patients.

Till now, the role of CRYAB in BC has not been reported. It is the first time that our study found CRYAB plays a vital role in diagnosis, metastasis and prognosis of HGBC patients. Both OS and DFS are worse in cases with lower CRYAB expression. CRYAB could enhance tumorigenesis by regulating the VEGF and conferring anti-VEGF resistance in breast cancer (20, 21). In addition, CRYAB participates in anti-apoptosis through activating the Akt signaling pathway, enhancing PI3K activity and inhibiting calcium-activated Raf/MEK/ERK signaling pathway (22–24). As a consequence, we hypothesized that CRYAB promotes tumorigenesis and resist cell apoptosis of HGBC via these signaling pathways. Subsequent GSEA analysis identified that CRYAB is associated with B cell receptor, T cell receptor, VEGF, MAPK, Wnt, and TGF-β signaling pathways, which supports our hypothesis and previous studies.

Previous investigation identified that upregulated ECM1 is associated with BC growth, migration, apoptosis and postoperative recurrence, which was in agreement with our results (25). However, the biological function of ECM1 in different tumors remains controversial. Wang' s study indicated that ECM1 might enhance cell proliferation and invasiveness by regulating the expression of glucose transporter 1, lactate dehydrogenase and hypoxia-inducible factor 1α (25). There is also evidence that ECM1 potentiates the phosphorylation of epidermal growth factor receptor (EGFR) and extracellular signal-regulated kinase through physical interaction with EGFR and activation of EGFR signaling in breast cancer development (26). Besides, studies based on pancreatic ductal adenocarcinoma suggested that increased ECM1 expression abrogated the anti-tumor effect exerted by miR-23a-5p (27). The present study indicated that ECM1 is as an independent prognostic indicator for HGBC and high ECM1 expression can also predict lymph node metastasis. Both GSEA and functional enrichments of ECM1 module showed that ECM1 expression is related to cell adhesion, extracellular matrix structural constituent and extracellular structure organization, which may be used to explain the metastasis of HGBC.

GPX3 is a member of a family of selenoproteins with vital antioxidant roles (28). It is reported that GPX3 is related to many malignancies including including head and neck, ovarian, and colon tumors (29, 30). Hypermethylation of the GPX3 promoter reduces GPX3 expression (31, 32). Furthermore, decreased GPX3 expression could inhibit clonogenicity and anchorage-independent cell survival in ovarian cancer progression (33). In addition, interactions between GPX3 and the p53-inducible gene 3 (PIG3) protein leads to activation of the apoptosis in prostate cancer cells (34). A retrospective study based on 40 BC patients reported that high GPX3 expression level in plasma might be predictive indicator for BC diagnosis and recurrence after transurethral resection (35). However, based on 405 BC samples, our results demonstrated that higher GPX3 expression level may predict an early UICC stage and better prognosis. Thus, the exact biological role of GPX3 and its potential mechanism for the progression and recurrence of BC are still unclear. Although our study contained a sufficient capacity, studies based on cells and a larger sample size are required to explore the relationship between GPX3 and BC. Functional enrichments showed that GPX3 plays a role in cellular detoxification and glutathione binding and metabolism. GSEA revealed that upregulated expression of GPX3 may act on extracellular matrix structure and positive regulation of vasculature development. We assumed that GPX3 is involved in the progression and recurrence of HGBC by participating in toxic metabolic process.

CGNL1, an endothelial junction complex protein, promotes GTPase mediated angiogenesis by strengthening adherens junctions via Rac1 activation, which further makes new blood vessels stable and extendable (36). What's more, CGNL1 is also involved in cell-cell junction assembly through regulating the activity of GTPases and Rac (37). Previous studies demonstrated that CGNL1 gene expression is associated with endometrial cancer survival (38). Our results showed that CGNL1 is a diagnostic factor for HGBC and can predict lymph node metastasis. GSEA and functional enrichments showed that CGNL1 may participate in HGBC progression by regulating cell-cell junction, tight junction, cytoskeletal protein binding, tropomyosin binding and growth factor binding, which confirms the findings of previous studies. However, further *in vitro* and *in vivo* studies are warranted to validate these mechanisms in BC.

Based on the networks of Bayesian analysis, we observed that the interaction of CRYAB and CGNL1 plays a key role in histological grade, UICC stage and pN stage of BC. Hence, we put CRYAB and CGNL1 into String online server to find the underlying mechanism of interaction. Functional enrichments showed that the two genes may have a combined effect on actin cytoskeleton (GO: 0015629, *P* = 0.039). In addition, GSEA of KEGG pathway analysis revealed that both genes are enriched in VEGF, MAPK, Wnt, and TGF-β signaling pathways.

Lymph node metastasis is a key indicator to predict poor prognosis of BC (39). In the present study, multivariate Cox regression showed that lymph node metastasis is an independent poor prognostic indicator of OS, which is consistent with previous research.

In the meantime, several limitations remained in our research. Firstly, although immunohistochemistry based on THPA and qRT-PCR based on urine samples confirmed the downregulation of CRYAB, ECM1, CGNL1, and GPX3 in HGBC and their diagnostic values, the exact molecular mechanisms of the these hub genes have not been investigated in the present study, and their prognostic values have not been proved by external validation. Secondly, immunohistochemistry was extracted from THPA. Even if we used Mann-Whitney U test and Fisher's Exact test to confirm the statistical significance, the number and information from THPA are still limited. Therefore, further studies based on a larger sample size and other racial types or regions are still required to verify these hypotheses and to make these results more convincible in the future.

## Conclusions

In general, our findings indicated that CRYAB, CGNL1, ECM1, and GPX3 are potential urine biomarkers of HGBC. All the four genes have the capability to be diagnostic indicators for HGBC. Furthermore, CRYAB, ECM1, and GPX3 are potential urine prognostic factors for HGBC, among which ECM1 and GPX3 might be considered as independent prognostic indicators for HGBC and new treatment targets as well. The four genes can also predict histological grade, UICC stage and lymph node metastasis. Immunohistochemistry and qRT-PCR were used to confirm the downregulation of the hub genes and their diagnostic values in HGBC. Among the four hub genes, CRYAB and CGNL1 have not been reported the relationship with HGBC before and the results of Bayesian analysis suggested that the interaction of CRYAB and CGNL1 plays a key role in HGBC. In addition, we used bioinformatics methods to explore the underlying mechanisms. These four novel urine biomarkers will have attractive applications to provide new diagnostic methods, prognostic predictors and treatment targets for HGBC, which could improve the prognosis of HGBC patients, if validated by further experiments and larger prospective clinical trials.

## Data Availability Statement

Publicly available datasets were analyzed in this study. The data can be found in Gene Expression Omnibus (GEO: http://www.ncbi.nlm.nih.gov/geo/), The Cancer Genome Atlas (TCGA: http://tcga-data.nci.nih.gov/tcga/), and The Human Protein Atlas (THPA: http://www.proteinatlas.org/). GSE68020 dataset was from GEO; BLCA (Bladder Urothelial Carcinoma) dataset was from TCGA; and immunohistochemistry was from THPA. All the data are open access. Summarized detailed characteristics of immunohistochemistry data from THPA are in Supplementary Table 1.

## Ethics Statement

The studies involving human participants were reviewed and approved by the Ethics Committee of Tianjin Medical University General Hospital. The patients/participants provided their written informed consent to participate in this study.

## Author Contributions

YS, JC, and DJ: research design. NO, YS, ZL, GC, and JC: data extraction and meta-analysis. YS, YY, and XL: drafting of the manuscript and modification.

### Conflict of Interest

The authors declare that the research was conducted in the absence of any commercial or financial relationships that could be construed as a potential conflict of interest.
