# Supplementary material for: Gene Expression Profiles Identified Novel Urine Biomarkers for Diagnosis and Prognosis of High-Grade Bladder Urothelial Carcinoma
Source: Front Oncol. 2020 Mar 27;10:394. doi: 10.3389/fonc.2020.00394 (PMC7118735; doi:10.3389/fonc.2020.00394)
Supplement: Supplementary file 1 [file Table_1.DOC]

**Supplementary Table 1.** Immunohistochemistry scores and characteristics from The Human Protein Atlas (THPA) database.

| Gene Name | Number | Type | Gender | Age | Tissue | Patient ID | Immunohistochemistry | | Score No.1 (percentage of the positive cells) | Score No.2 (intensity of the cellular staining) | Total staining score (Score No.1*Score No.2) | Expression Level |
| --- | --- | --- | --- | --- | --- | --- | --- | --- | --- | --- | --- | --- |
|  |  |  |  |  |  |  | Intensity | Quantity |  |  |  |  |
| ECM1 |  |  |  |  |  |  |  |  |  |  |  |  |
|  | Control 1 | Normal Urinary bladder | Male | 76 | Normal tissue, NOS (M-00100) | 4790 | Mild | 5%-25% | 1 | 1 | 1 | + |
|  | Control 2 | Normal Urinary bladder | Male | 55 | Normal tissue, NOS (M-00100) | 751 | Mild | 5%-25% | 1 | 1 | 1 | + |
|  | Case 1 | Bladder urothelial cancer | Female | 60 | Urothelial carcinoma, High grade (M-812033) | 3517 | Absent | <5% | 0 | 0 | 0 | - |
|  | Case 2 | Bladder urothelial cancer | Male | 67 | Urothelial carcinoma, High grade (M-812033) | 5000 | Absent | <5% | 0 | 0 | 0 | - |
|  | Case 3 | Bladder urothelial cancer | Male | 57 | Urothelial carcinoma, High grade (M-812033) | 1906 | Absent | <5% | 0 | 0 | 0 | - |
|  | Case 4 | Bladder urothelial cancer | Male | 56 | Urothelial carcinoma, High grade (M-812033) | 1760 | Absent | <5% | 0 | 0 | 0 | - |
|  | Case 5 | Bladder urothelial cancer | Female | 63 | Urothelial carcinoma, High grade (M-812033) | 3516 | Absent | <5% | 0 | 0 | 0 | - |
|  | Case 6 | Bladder urothelial cancer | Female | 70 | Urothelial carcinoma, High grade (M-812033) | 3079 | Absent | <5% | 0 | 0 | 0 | - |
|  | Case 7 | Bladder urothelial cancer | Female | 85 | Urothelial carcinoma, High grade (M-812033) | 1871 | Absent | <5% | 0 | 0 | 0 | - |
|  | Case 8 | Bladder urothelial cancer | Female | 78 | Urothelial carcinoma, High grade (M-812033) | 3111 | Absent | <5% | 0 | 0 | 0 | - |
|  | Case 9 | Bladder urothelial cancer | Female | 49 | Urothelial carcinoma, High grade (M-812033) | 4948 | Absent | <5% | 0 | 0 | 0 | - |
| GPX3 |  |  |  |  |  |  |  |  |  |  |  |  |
|  | Control 1 | Normal Urinary bladder | Male | 66 | Normal tissue, NOS (M-00100) | 1824 | Mild | 51%-75% | 3 | 1 | 3 | + |
|  | Control 2 | Normal Urinary bladder | Male | 76 | Normal tissue, NOS (M-00100) | 4790 | Mild | 51%-75% | 3 | 1 | 3 | + |
|  | Case 1 | Bladder urothelial cancer | Male | 76 | Urothelial carcinoma, High grade (M-812033) | 4947 | Absent | <5% | 0 | 0 | 0 | - |
|  | Case 2 | Bladder urothelial cancer | Male | 60 | Urothelial carcinoma, High grade (M-812033) | 4937 | Absent | <5% | 0 | 0 | 0 | - |
|  | Case 3 | Bladder urothelial cancer | Male | 79 | Urothelial carcinoma, High grade (M-812033) | 4949 | Mild | 51%-75% | 3 | 1 | 3 | + |
|  | Case 4 | Bladder urothelial cancer | Female | 58 | Urothelial carcinoma, High grade (M-812033) | 4543 | Absent | <5% | 0 | 0 | 0 | - |
|  | Case 5 | Bladder urothelial cancer | Female | 70 | Urothelial carcinoma, High grade (M-812033) | 3079 | Absent | <5% | 0 | 0 | 0 | - |
|  | Case 6 | Bladder urothelial cancer | Female | 85 | Urothelial carcinoma, High grade (M-812033) | 1871 | Absent | <5% | 0 | 0 | 0 | - |
|  | Case 7 | Bladder urothelial cancer | Female | 78 | Urothelial carcinoma, High grade (M-812033) | 4934 | Absent | <5% | 0 | 0 | 0 | - |
|  | Case 8 | Bladder urothelial cancer | Male | 62 | Urothelial carcinoma, High grade (M-812033) | 4946 | Absent | <5% | 0 | 0 | 0 | - |
| CRYAB |  |  |  |  |  |  |  |  |  |  |  |  |
|  | Control 1 | Normal Urinary bladder | Male | 66 | Normal tissue, NOS (M-00100) | 1824 | Absent | <5% | 0 | 0 | 0 | - |
|  | Control 2 | Normal Urinary bladder | Male | 76 | Normal tissue, NOS (M-00100) | 4790 | Absent | <5% | 0 | 0 | 0 | - |
|  | Control 3 | Normal Urinary bladder | Male | 66 | Normal tissue, NOS (M-00100) | 1824 | Absent | <5% | 0 | 0 | 0 | - |
|  | Control 4 | Normal Urinary bladder | Male | 76 | Normal tissue, NOS (M-00100) | 4790 | Absent | <5% | 0 | 0 | 0 | - |
|  | Control 5 | Normal Urinary bladder | Male | 66 | Normal tissue, NOS (M-00100) | 1824 | Absent | <5% | 0 | 0 | 0 | - |
|  | Case 1 | Bladder urothelial cancer | Female | 63 | Urothelial carcinoma, High grade (M-812033) | 3516 | Absent | <5% | 0 | 0 | 0 | - |
|  | Case 2 | Bladder urothelial cancer | Female | 56 | Urothelial carcinoma, High grade (M-812033) | 3465 | Absent | <5% | 0 | 0 | 0 | - |
|  | Case 3 | Bladder urothelial cancer | Male | 56 | Urothelial carcinoma, High grade (M-812033) | 1760 | Absent | <5% | 0 | 0 | 0 | - |
|  | Case 4 | Bladder urothelial cancer | Male | 59 | Urothelial carcinoma, High grade (M-812033) | 5575 | Absent | <5% | 0 | 0 | 0 | - |
|  | Case 5 | Bladder urothelial cancer | Female | 79 | Urothelial carcinoma, High grade (M-812033) | 4718 | Absent | <5% | 0 | 0 | 0 | - |
|  | Case 6 | Bladder urothelial cancer | Male | 46 | Urothelial carcinoma, High grade (M-812033) | 3184 | Absent | <5% | 0 | 0 | 0 | - |
|  | Case 7 | Bladder urothelial cancer | Female | 85 | Urothelial carcinoma, High grade (M-812033) | 1871 | Absent | <5% | 0 | 0 | 0 | - |
|  | Case 8 | Bladder urothelial cancer | Female | 41 | Urothelial carcinoma, High grade (M-812033) | 3473 | Marked | 51%-75% | 3 | 3 | 9 | +++ |
|  | Case 9 | Bladder urothelial cancer | Male | 35 | Urothelial carcinoma, High grade (M-812033) | 2311 | Absent | <5% | 0 | 0 | 0 | - |
| CGNL1 |  |  |  |  |  |  |  |  |  |  |  |  |
|  | Control 1 | Normal Urinary bladder | Male | 51 | Normal tissue, NOS (M-00100) | 1761 | Absent | <5% | 0 | 0 | 0 | - |
|  | Control 2 | Normal Urinary bladder | Male | 57 | Normal tissue, NOS (M-00100) | 1906 | Absent | <5% | 0 | 0 | 0 | - |
|  | Control 3 | Normal Urinary bladder | Male | 55 | Normal tissue, NOS (M-00100) | 3316 | Absent | <5% | 0 | 0 | 0 | - |
|  | Case 1 | Bladder urothelial cancer | Female | 79 | Urothelial carcinoma, High grade (M-812033) | 3265 | Absent | <5% | 0 | 0 | 0 | - |
|  | Case 2 | Bladder urothelial cancer | Female | 60 | Urothelial carcinoma, High grade (M-812033) | 3517 | Absent | <5% | 0 | 0 | 0 | - |
|  | Case 3 | Bladder urothelial cancer | Male | 64 | Urothelial carcinoma, High grade (M-812033) | 2839 | Absent | <5% | 0 | 0 | 0 | - |
|  | Case 4 | Bladder urothelial cancer | Male | 89 | Urothelial carcinoma, High grade (M-812033) | 2704 | Absent | <5% | 0 | 0 | 0 | - |
|  | Case 5 | Bladder urothelial cancer | Female | 70 | Urothelial carcinoma, High grade (M-812033) | 3079 | Absent | <5% | 0 | 0 | 0 | - |
|  | Case 6 | Bladder urothelial cancer | Male | 50 | Urothelial carcinoma, High grade (M-812033) | 1717 | Absent | <5% | 0 | 0 | 0 | - |
|  | Case 7 | Bladder urothelial cancer | Male | 67 | Urothelial carcinoma, High grade (M-812033) | 810 | Absent | <5% | 0 | 0 | 0 | - |
|  | Case 8 | Bladder urothelial cancer | Female | 85 | Urothelial carcinoma, High grade (M-812033) | 1871 | Absent | <5% | 0 | 0 | 0 | - |
|  | Case 9 | Bladder urothelial cancer | Female | 63 | Urothelial carcinoma, High grade (M-812033) | 3516 | Mild | 5%-25% | 1 | 1 | 1 | + |
|  | Case 10 | Bladder urothelial cancer | Female | 75 | Urothelial carcinoma, High grade (M-812033) | 2699 | Absent | <5% | 0 | 0 | 0 | - |
| CRNN |  |  |  |  |  |  |  |  |  |  |  |  |
|  | Control 1 | Normal Urinary bladder | Female | 62 | Normal tissue, NOS (M-00100) | 3754 | Absent | <5% | 0 | 0 | 0 | - |
|  | Control 2 | Normal Urinary bladder | Male | 83 | Normal tissue, NOS (M-00100) | 1870 | Absent | <5% | 0 | 0 | 0 | - |
|  | Control 3 | Normal Urinary bladder | Female | 62 | Normal tissue, NOS (M-00100) | 3754 | Mild | 76%-100% | 4 | 1 | 4 | + |
|  | Control 4 | Normal Urinary bladder | Male | 83 | Normal tissue, NOS (M-00100) | 1870 | Mild | 76%-100% | 4 | 1 | 4 | + |
|  | Case 1 | Bladder urothelial cancer | Female | 60 | Urothelial carcinoma, High grade (M-812033) | 3517 | Mild | 5%-25% | 1 | 1 | 1 | + |
|  | Case 2 | Bladder urothelial cancer | Female | 82 | Urothelial carcinoma, High grade (M-812033) | 3112 | Absent | <5% | 0 | 0 | 0 | - |
|  | Case 3 | Bladder urothelial cancer | Female | 80 | Urothelial carcinoma, High grade (M-812033) | 2031 | Absent | <5% | 0 | 0 | 0 | - |
|  | Case 4 | Bladder urothelial cancer | Male | 50 | Urothelial carcinoma, High grade (M-812033) | 1717 | Mild | 5%-25% | 1 | 1 | 1 | + |
|  | Case 5 | Bladder urothelial cancer | Male | 35 | Urothelial carcinoma, High grade (M-812033) | 2311 | Absent | <5% | 0 | 0 | 0 | - |
|  | Case 6 | Bladder urothelial cancer | Female | 85 | Urothelial carcinoma, High grade (M-812033) | 1871 | Absent | <5% | 0 | 0 | 0 | - |
|  | Case 7 | Bladder urothelial cancer | Male | 51 | Urothelial carcinoma, High grade (M-812033) | 2053 | Absent | <5% | 0 | 0 | 0 | - |

**Note.** Staining intensity was scored as follows: absent staining, 0; mild staining, 1; moderate staining, 2; marked staining, 3. Percentages of positive cells were categorized as follows: < 5% of positive cells, 0; 5%‐25%, 1; 26%‐50%, 2; 51%‐75%, 3; 76%‐100%, 4. For each case, the two scores were multiplied to produce a total staining score. According to the total staining scores, we divided the expression into four levels: negative (-, score 0); weakly positive (+, scores 1‐4); positive (++, scores 5-8); strongly positive (+++, scores 9-12).
